# Statistical Modeling of Lung Cancer: Answering Relative Questions

**Published:** 2011-03

**Authors:** Chunling Cong, James Kepner, Chris. P. Tsokos

**Affiliations:** 1 *Department of Mathematics and Statistics, University of South Florida, Tampa, FL, USA;*; 2 *American Cancer Society, Atlanta, USA*

**Keywords:** lung cancer, parametric, nonparametric, accelerated failure time, survival analysis

## Abstract

The objective of this paper is to perform parametric and nonparametric analysis to address some very important questions concerning lung cancer utilizing real lung cancer data: What is the probabilistic nature of mortality time in ex-smoker lung cancer patients and non-smoker lung cancer patients, for female, male, and the totality of female and male patients? Is there significant difference of mortality time between ex-smoker and non-smoker patients? For ex-smokers, are there any differences with respect to the key variables such as mortality time, cigarettes per day (CPD), and duration of smoking between female and male patients? For non-smokers, can we notice a difference in mortality time between female and male patients? Can we accurately predict mortality time given information on CPD, starting time and quitting time for a specific lung cancer patient who smokes? Thus best fitting probability distributions are identified and their parameters are estimated. Mean mortality times are compared between non-smokers and ex-smokers, female non-smokers and male non-smokers, and female ex-smokers and male ex-smokers. Important entities related to lung cancer mortality time, such as cigarettes per day (CPD), and duration of smoking (DUR), are compared between female and male ex-smoker lung cancer patients. Finally, a model is developed to predict the mortality time of ex-smokers with a high degree of accuracy.

## INTRODUCTION

Lung cancer is a disease of uncontrolled cell growth in tissues of the lung and one of the deadliest common cancers in both men and women. Annually, 1.3 million deaths are caused by lung cancer worldwide. It is more common in older adults than in people under age 45. It is known that cigarette smoking is the leading cause of lung cancer, which means the risk of getting lung cancer is strongly associated with the number of cigarettes smoked per day and the time when one starts and quits smoking. Secondhand smoke contributes to lung cancer as well and there is a chance that people who have never smoked will get lung cancer. More information on lung cancer can be found in (1-6).

## MATERIALS AND METHODS

The data that were first collected in 1982 and the mortality follow-up in the dataset is complete through 2006. It encompasses 1.2 million subjects in 50 states. Only data from patients with lung cancer are included in this study. For ex-smokers, the total number of lung cancer patients is 5,316, of which 1,523 are females and 3,793 are males. For non-smokers, the total number of lung cancer patients is 2,010, of which 1,386 are females and 624 are males.

**Figure 1:**
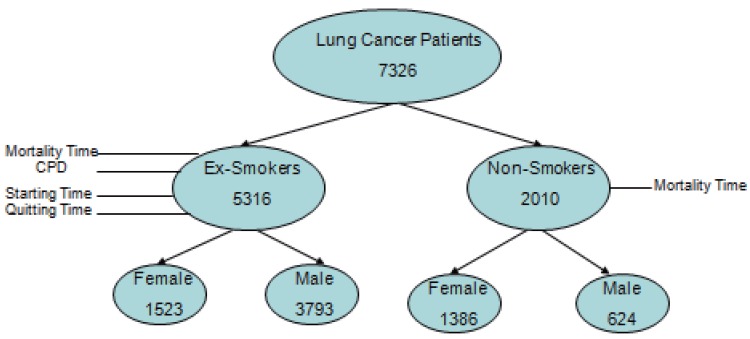
Lung Cancer Data.

Although there are many other causes associated with lung cancer such as air pollution, radon gas, asbestos, family history of lung cancer, radiation therapy to the lungs, and exposure to cancer-causing chemicals, we confined our interest in smoking only due to the lack of data pertaining to these causes. The four variables of interest are the number of cigarettes per day (CPD), the age at which an individual started smoking (*t_s_*), the age at which an individual quit smoking (*t_q_*), smoking duration (in years), *t_q_*–*t_s_* (DUR), and mortality time (*t_m_*). The following diagram gives a clearer view of what the data looks like (Figure 1).

The objective of this study is to address the following questions related to some of the most important entities in lung cancer: cigarettes per day (CPD), time the patient started smoking (*t_s_*), time the patient quit smoking (*t_q_*), duration of smoking which is defined as the difference between the two above mentioned times (DUR), and, most importantly, the mortality time (*t_m_*):

- What is the probabilistic nature of mortality time in ex-smoker lung cancer patients and non-smoker lung cancer patients, for female, male, and the totality of female and male patients?
- Is there significant difference of mortality time between ex-smoker and non-smoker patients?
- For ex-smokers, are there any differences with respect to the key variables such as mortality time, CPD, and duration of smoking between female and male patients?
- For non-smokers, can we notice a difference in mortality time between female and male patients?
- Can we accurately predict mortality time given information on CPD, starting time and quitting time for a specific lung cancer patient who smokes?

## RESULTS OF PARAMETRIC ANALYSIS

Before modeling mortality time as a function of CPD, DUR, *t_s_*, *t_q_*, basic parametric analysis should be performed to understand its probabilistic behavior. More than 40 different classical distributions are fit to the data and three goodness-of-fit tests, Kolmogorov-Smirnov (7), Anderson-Darling (8) and Chi-Square (9, 10) are conducted for the mortality time of lung cancer patients for female ex-smokers, male ex-smokers, all ex-smokers, female non-smokers, male non-smokers, all non-smokers, respectively. The parameters of the three distributions ranked the highest by goodness-of-fit are listed and the 90% and 95% confidence intervals are constructed. The results appear in the following tables where Table 1 shows the mean and variance of the best fitting distribution and Table 2 shows the 90% and 95% confidence bands of the true mean of the estimated distributions.

**Table 1:** Mean and Standard Deviation of Fitted Distributions

	Johnson SB	Beta	Three-parameter Weibulll

Female ex-smokers	NA	73.995 (8.9577)	74.007 (8.9365)
Male ex-smokers	NA	74.543 (8.1875)	74.542 (8.2119)
Ex-smokers	NA	74.384 (8.4155)	74.387 (8.428)
Female non-smokers	NA	76.117 (10.213)	76.148 (10.165)
Male non-smokers	NA	76.011 (9.6368)	76.015 (9.6551)
Non-smokers	NA	76.085 (10.041)	76.103 (10.022)

Due to the complexity of the calculation of Johnson SB, the mean and variance were not obtained as output through statistical software.

**Table 2:** 90% and 95% Confidence Interval of the True Mean

	Johnson SB	Beta	Three-parameter Weibulll

Female ex-smokers	(59.9, 87.622) (55.419, 90.07)	(58.586, 88.016) (55.364, 90.327)	(58.456, 87.916) (55.371, 90.168)
Male ex-smokers	(60.527, 87.493) (57.827, 89.55)	(60.557, 87.52) (57.849, 89.591)	(60.304, 87.386) (57.519, 89.482)
Ex-smokers	(59.9, 87.622) (57.061, 89.701)	(59.98, 87.659) (57.057, 89.85)	(59.751, 87.541) (56.871, 89.68)
Female non-smokers	(58.145, 91.777) (54.682, 93.933)	(58.304, 91.886) (54.794, 94.142)	(58.277, 91.742) (54.582, 94.205)
Male non-smokers	(58.888, 90.419) (55.014, 92.541)	(58.87, 90.391) (55.045, 92.434)	(58.671, 90.298) (54.727, 92.425)
Non-smokers	(58.367, 91.373) (54.761, 93.541)	(58.461, 91.428) (54.811, 93.643)	(58.354, 91.302) (54.567, 93.657)

As can be seen from the above tables, the best fitting distribution is always Johnson SB followed by Beta distribution and three-parameter Weibull distribution. With this finding, we can find the mean and variance and construct the 90% and 95% confidence intervals for mortality time. An interesting thing worth noting is that no matter which distribution is chosen, Beta, Johnson SB, or three-parameter Weibull, their 90% and 95% confidence intervals are very close. Although Johnson SB appears to be the best fit for both female and male ex-smokers and theoretically a likelihood ratio test could be applied to test the difference of means of mortality time in these two groups, the parametric comparison is not used here due to the extremely complicated calculation. Furthermore, it appears that there are no significant differences between the means and variances of mortality times for females and males. However, it is observed that ex-smoker lung cancer patients have decreased mortality compared to non-smoker lung cancer patients. These parametric results lead us to compare the key variables in the different groups, between female and males, or between non-smokers and ex-smokers using non-parametric methods as described next.

## RESULTS OF NONPARAMETRIC COMPARISON

After finding the mortality time for both ex-smokers and non-smokers, the next question is whether there is significant difference of mortality time between ex-smokers and non-smokers, between female and male groups. We are also interested in the impact of the number of cigarettes smoked per day and duration of smoking on female and male smokers (for ex-smokers only since they are all zeros for non-smokers). The Wilcoxon Rank Sum two - sample test (11, 12) was performed to detect location differences. The results are shown in Table 3. For all these tests of hypothesis, we first set the null hypothesis to be two-sided, if *p*-value is large enough; we fail to reject the null hypothesis. However, if *p*-value is small which suggests the rejection of the null hypothesis, we proceed to test the one-sided hypothesis.

**Table 3:** Wilcoxon Two-Sample Test Results

*H_o_*	*t_m(ex)_ = t_m(non)_*	*t_m(female-ex)_ = t_m(male-ex)_*	*t_m(female-non)_ = t_m(male-non)_*	*CPD_male_ ≤ CPD_female_*	*DUR_male_ ≤ DUR_female_*

*p*-value	0.0018	0.1180	0.8106	<0.0001	0.0001
Conclusion	Reject	Accept	Accept	Reject	Reject

### Mortality time between ex-smokers and non-smokers

Mortality time of ex-smokers and nonsmokers are compared using the Wilcoxon two-sample test. Under hypothesis that *t_m(ex-smokers)_* ≥ *t_m(non-smokers)_*, the *p*-value is 0.0018. Thus, using a significance level of 0.05, we reject the null hypothesis and conclude that non-smoker lung cancer patients have longer mortality time than that of ex-smokers.

### Ex-Smokers mortality time between female and male

There is no significant difference between the female and male smokers with respect to the death time from lung cancer. For two-sided hypothesis, the *p*-value is 0.1180 and the *p*-value for one-sided hypothesis is 0.0590 which is still higher than 0.05. Thus, using a significance level of 0.05, death time of female ex-smokers is not significantly different from that of male ex-smokers (*p*=0.1180).

### Non-Smokers mortality time between female and male

As can be seen from the two-sided *p*-value 0.8106 and one-sided *p*-value of 0.4053, there is insufficient evidence to conclude that there is a difference between female and male non-smoker lung cancer patients. Thus, no difference of mortality time can be found between female and male lung cancer patients, both in ex-smokers and non-smokers, which is consistent with the conclusion from parametric analysis.

### Ex-smokers CPD

As can be seen from the *p*-value, which is less than 0.0001 under the hypothesis that *CPD_male_* ≤ *CPD_female_*, there is strong evidence that males tend to have more cigarettes per day than females.

### Ex-smokers DUR

Similarly, the *p*-value of 0.0001 under null hypothesis that *DUR_male_* ≤ *DUR_female_* suggests that smoking duration for male smokers exceeds the smoking duration of female smokers.

In summarizing the above analyses, the following conclusions are obtained:

- There is no significant difference in mean mortality time for females and males for both ex-smokers and non-smokers. Ex-smokers tend to have a shorter mortality time than non-smokers.
- For CPD, mean CPD of males is larger than that of females.
- For DUR, males have longer duration of smoking than females.

## RESULTS OF MODELING OF MORTALITY TIME

After finding the probabilistic behavior of mortality time and comparison of key entities with respect to gender and smoking status, we proceed to investigate the relation between mortality time and other attributable variables such as *CPD*, time an individual started smoking (*t_s_*), and time an individual quit smoking (*t_q_*), where multiple regression models is most commonly used tool. First, for the female ex-smokers, multiple regression models were run and the backward selection method is used to eliminate any variables that do not significantly contribute. However, after multiple regression is applied using mortality time as the response variable and *CPD*, *t_s_*, *t_q_*, and the second-order interaction between them as well as the quadratic terms, the R-square (0.2249) of the full model is pretty small which indicates multiple regression model is not a good choice here. The same procedure was applied to male ex-smokers, where the R-square was only 0.1301.

Although multiple regression models do not perform well, they give us some guidance on which variables are not important and can be eliminated in the modeling process later. We then proceeded to utilize the survival regression model, also called the accelerated failure time (AFT) model, which assumes certain distribution of the response variables.

### AFT model

When covariates are considered, we assume that the relapse time has explicit relationships with the covariates. Furthermore, when a parametric model is considered, we assume that the relapse time follows a given theoretical probability distribution and has an explicit relationship with the covariates.

Let* T* denote a continuous non-negative random variable representing the survival time (relapse time in this case), with probability density function (pdf) *f (t)* and cumulative distribution (cdf) *F (t)* = Pr (T≤t). We will focus on the survival function *S (t)* = Pr (T>t), the probability of being alive at *t*. In this model, we start from a random variable *W* with a standard distribution in (-∞, +∞) and generate a family of survival distributions by introducing location and scale parameters to relate to the relapse time as follows:

(a) Y=log T=a+σ W

Where α and σ are the location and scale parameters, respectively.

Adding covariates into the location parameter in equation (a) we have

(b) Y=log T=xβ+σ W

where the error term *W* has a suitable probability distribution, e.g. extreme value, normal or logistic. This transformation leads to the Weibull, log-normal and log-logistic models for *T*. This type of statistical model is called an accelerated failure time (AFT) model.

Other information on AFT models can be found in (13-15).

### Females

Survival regression models were run using statistical software including exponential, generalized gamma, loglogistic, lognormal, logistic, normal, and Weibull distributions. Their log likelihoods were: -1532, 1215, 1189, 1194, -5326, -5309, 1185. Thus, the generalized gamma was determined to prove the best fit and backward elimination was used to eliminate the unimportant variables. In the final model, the variables left are CPD, *t_s_*, interaction between *t_s_* and* t_q_*, interaction between CPD and *t_q_*, and quadratic terms of CPD and* t_q_*.

All terms in the model are significant and they are ranked according to their significance. The quadratic term of quitting time ranks first followed by CPD, starting time, interaction between starting time and quitting time, quadratic term of CPD, and interaction between CPD and quitting time.

The following percentage plot is obtained for the final model.

After the estimations of the parameters in the model are obtained, the value of log (T) can be predicted by plugging the parameters into the equation, and thus mortality time T can be calculated by simply taking natural exponentials. The mean and standard deviation of the difference between predicted mortality time and observed mortality time are 0.1378148 and 7.911363, respectively. However, the mean and variance of the difference between predicted log (T) and observed log (T) (residual) are only 0.008175027 and 0.1108183, respectively. Figure 3 shows the survival curves constructed by predicted mortality time and observed mortality time.

**Figure 2:**
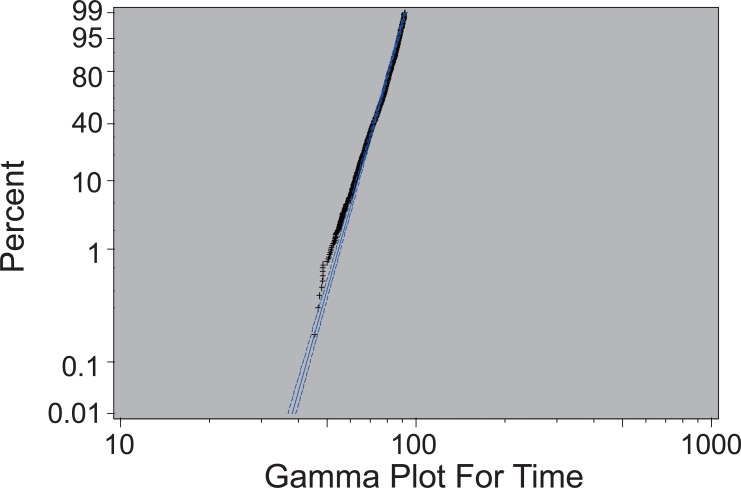
Percentage Plot for Female Ex-Smokers.

After elimination of the insignificant variables, all the variables left in the models are significant and the loglikelihood is not much changed. As can be observed from the Figure 2, all the data falls within the 95% confidence interval of the estimated percentage except in the left tail which suggests the model is fairly accurate.

**Figure 3:**
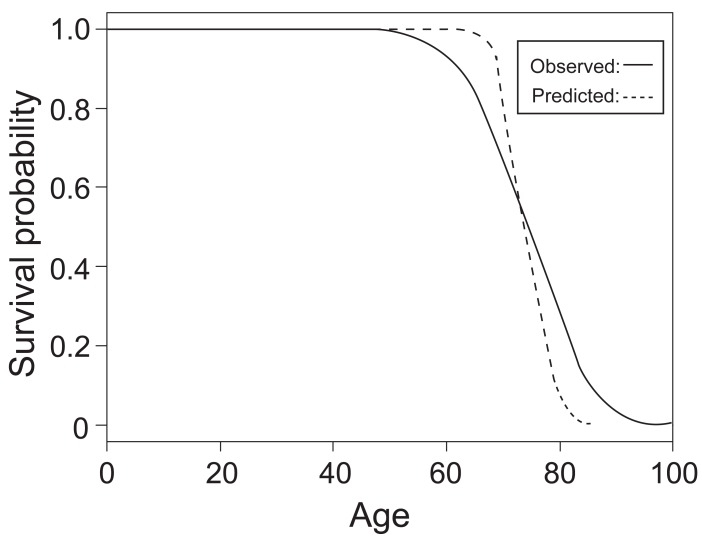
Survival Curves from Predicted and Observed Mortality Time.

### Males

The same procedure is followed for male ex-smoker lung cancer patients. Survival regression models were run including exponential, gamma, loglogistic, lognormal, logistic, normal, and Weibull. And their log likelihoods are as follows: -3814, 3209, 3128, 3160, -13152, -13093, 3121. Thus, the three parameter gamma is chosen to be the best fitting distribution and backward elimination is used to eliminate interactions between CPD and *t_s_*, *t_s_* and *t_q_*, and the quadratic term of *t_s_*. In the final model, the variables left are CPD, *t_s_*, *t_q_*, interaction between CPD and *t_q_*, and quadratic terms of CPD and *t_q_*.

All the terms are significant, and quadratic term of quitting time ranks first followed by quitting time, CPD, starting time, interaction between CPD and quitting time, and quadratic term of CPD comes last.

Similarly, after plugging the estimated parameters into the model and obtaining the value of log (T), mortality time T can be easily calculated by taking natural exponentials. The mean and standard deviation of the difference between predicted mortality time and observed mortality time are 0.1439845 and 7.651358, respectively. However, since the model is constructed using log (T) as the response variable, the mean and variance of the difference between predicted log (T) and observed log (T) are only 0.007539421 and 0.1054347, respectively, which indicates the predictive power of the model. Figure 5 below shows the survival curves constructed by predicted mortality time and observed mortality time.

**Figure 4:**
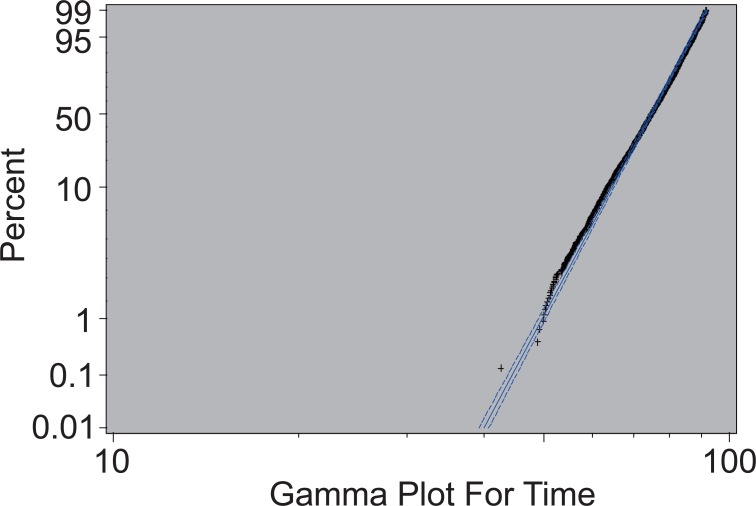
Percentage Plot of Male Ex-Smokers.

As can be observed from Figure 4, the percentage calculated from real data falls well within the 95% confidence interval constructed from the mode. This suggests the model is fairly accurate.

**Figure 5:**
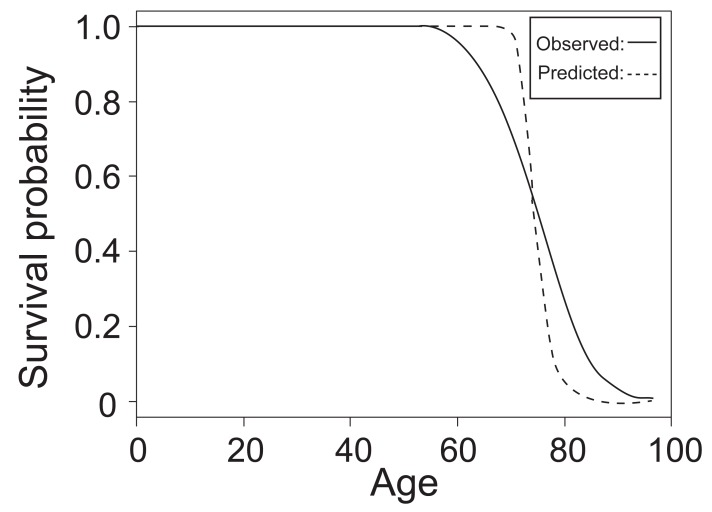
Survival Curves from Predicted and Observed Mortality Time.

## DISCUSSION

By using parametric analysis, distributions of mortality time for both female and male ex-smokers and non-smokers are found. Ninety percent and 95% confidence intervals are constructed which provide basic information on the probabilistic behavior of mortality time. Using nonparametric methods, we found that there is no significant difference in mean mortality time for females and males for both ex-smokers and non-smokers. Ex-smokers tend to have a shorter mortality time than non-smokers; mean CPD of males is larger than that of females; males have longer duration of smoking than females. Lastly, an accelerated failure time model is constructed for female and male lung cancer patients, respectively, so that given information on cigarettes per day, time started smoking, and time quit smoking of a specific smoker, mortality time can be predicted.
